# The Passing of the Automobile

**Published:** 1899-12

**Authors:** 


### THE PASSING OF THE AUTOMOBILE.

Those New York dailies that have been slopping over in their zeal to boom the automobile speculative craze are now breaking their necks to get in touch with what they are disposed to term the new craze for horses. If a small part of their zeal and labors during the past two years in booming and anticipating a horseless age had been given to advance and promote the horse industry, a legitimate field of investment, they might have the one great consolation of having served a valued source of wealth in our land, an industry that has promoted the welfare of our people in every station and condition of life, a field where man has found the keenest pleasure and companionship that can never be found associated with so inanimate and unattractive a machine as fills the rôle to-day of an automobile.

The Veterinary Medical Association of New Jersey will hold, in Newark, on January 11, 1900, a very important meeting. This organization at its last gathering added material strength to its membership-list, and there is a strong disposition manifest to make one vigorous organization throughout the State, and to make itself felt in gaining greater and better recognition for the profession, in moulding legislation on lines of kindred interest to the citizens of that State and the veterinary profession, more in touch with the advanced thoughts of the day, and to better the protection of the livestock owners from the losses that fall heavily upon their overburdened shoulders. The Journal looks with pleasure upon this movement to combine the strength of the profession, and will aid in every way this promised movement. Its columns are open to its support, and with a knowledge of how strong the profession is individually throughout that Commonwealth, we bespeak a new era of progress and advancement all along the line for our co-workers in New Jersey. Let the whole profession give a helping hand.

